# Novel Biomarkers and Imaging Tests for Acute Kidney Injury Diagnosis in Patients with Cancer

**DOI:** 10.34067/KID.0000000660

**Published:** 2024-11-21

**Authors:** Kavita Mistry, Sagar Sadarangani, Daiana Moreno, Sherley M. Mejia, Dennis G. Moledina, Meghan E. Sise

**Affiliations:** 1 Division of Nephrology, Department of Medicine, Beth Israel Deaconess Medical Center and Harvard Medical School, Boston, Massachusetts; 2 Section of Nephrology, Department of Internal Medicine, Yale School of Medicine, New Haven, Connecticut; 3 Division of Nephrology, Department of Medicine, Massachusetts General Hospital, Boston, Massachusetts; 4 Department of Internal Medicine, Clinical and Translational Research Accelerator, New Haven, Connecticut

**Keywords:** AKI, cancer, biomarkers, onconephrology

## Abstract

The lack of noninvasive urine and blood-based biomarkers for the diagnosis of AKI in patients with cancer is an area of significant unmet clinical need. Traditional noninvasive diagnostic tools that are currently used in the clinic, such as creatinine and cystatin C–based eGFR measurements, urinalysis, urine sediment examination, urine protein quantification, and urine electrolyte measurement, lack the sensitivity and specificity to distinguish between the various underlying etiologies of AKI in patients with cancer. Imaging-based diagnostics can be helpful to rule out urinary obstruction, but also lack sensitivity and specificity to diagnose the etiology of AKI. Kidney biopsy is often required for definitive diagnosis. As our scientific understanding of the biological pathways that are dysregulated in AKI has advanced, there has been considerable interest in developing new biomarkers for AKI. For example, the diagnosis of acute interstitial nephritis, which can occur in patients treated with immune checkpoint inhibitors, promises to be revolutionized by the incorporation of urinary testing for inflammatory biomarkers, such as C-X-C motif ligand 9, TNF-*α*, and IL-9. In the case of cisplatin administration, biomarkers such as neutrophil gelatinase-associated lipocalin and kidney injury molecule-1 may improve prognostication, differentiating between persistent AKI resulting from acute tubular injury versus prerenal azotemia. The development and validation of blood, urine, and imaging biomarkers into widely used diagnostic tests will require a concerted effort, but could improve diagnosis, management, and prognostication for a growing group of patients who are at high risk of developing AKI during the course of their illness.

## Introduction

AKI is common in patients with cancer, affecting 15%–25% of patients receiving systemic antineoplastic therapies.^1^ AKI is a heterogeneous condition, triggered by a wide array of insults, ranging in severity from subclinical to severe anuric kidney failure, and characterized by a diverse set of underlying pathological processes. Despite significant advancements in the understanding of the causes and molecular mechanisms of AKI, the routine diagnosis and management of AKI in the clinical setting continues to suffer from a lack of translation of new scientific discoveries into operational clinical tools. Kidney biopsy remains the gold standard for diagnosis; however, it is used in a minority of patients because of heightened risks in patients with cancer.^2–4^ Particularly, in patients with cancer, where AKI can affect a patient's ability to continue antineoplastic therapy or to enroll in a clinical trial, there is an urgent need for improved noninvasive diagnostics.

In this review, we describe how traditional biomarkers of AKI are used for diagnosis and monitoring in patients with cancer, as well as how novel and emerging blood, urine, and imaging tests may facilitate noninvasive diagnosis of AKI. In particular, we highlight the growing body of research on biomarkers of acute interstitial nephritis (AIN), a disease that presents unique opportunities for the development of novel diagnostics.

## Multifactorial Causes of AKI in Patients with Cancer

**Figure 1:**
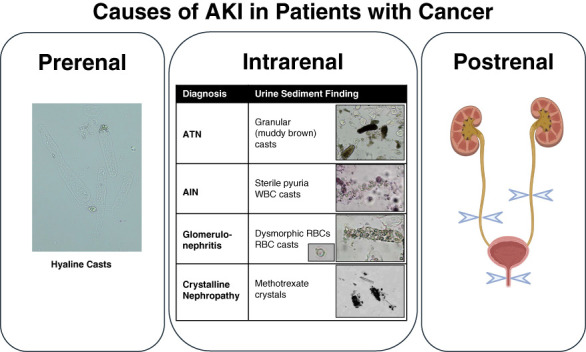
**Causes of AKI in patients with cancer.** The differential diagnosis of AKI in patients with cancer encompasses prerenal, intrarenal, and postrenal etiologies. Classic urine sediment findings that can be helpful in the diagnosis of AKI include hyaline casts that can be seen in prerenal azotemia, granular (muddy brown) casts that can be seen in ATN, sterile pyuria and WBC casts that are a hallmark of AIN, and dysmorphic RBCs and RBC casts that occur in GN. Crystals may also be observed in patients with crystalline nephropathy, as with high-dose methotrexate. The diagnosis of postrenal AKI requires imaging (*e.g*., ultrasound and cross-sectional imaging). AIN, acute interstitial nephritis; ATN, acute tubular necrosis; RBC, red blood cell; WBC, white blood cell. Created in BioRender. Mistry *et al.*^8^ BioRender.com/v78p512.

The differential diagnosis of AKI in patients with cancer is broad (Figure 1) and encompasses prerenal, intrinsic, and postrenal etiologies.^5,6^ Prerenal AKI occurs as a sequela of chemotherapy side effects, such as anorexia, nausea, vomiting, and diarrhea, that decrease plasma volume and reduce blood flow to the kidneys. Patients with decompensated cirrhosis in the setting of malignancy may also develop prerenal AKI because of low effective circulating volume. Intrinsic AKI due to acute tubular necrosis (ATN) may occur in severe cases of volume depletion or sepsis or may be a direct complication of cancer or its treatment.^7–11^ Causes of intrinsic AKI directly attributable to malignancies include cast nephropathy in multiple myeloma, lymphomatous kidney infiltration, and paraneoplastic glomerular diseases.^12,13^ Intrinsic AKI due to tubular cell, podocyte, or endothelial cell injury can also occur after traditional chemotherapies, immunotherapies, cellular therapies, or targeted therapies (Table 1). Immune checkpoint inhibitors (ICIs) can lead to AKI by causing AIN, particularly when used in combination with other medications that trigger AIN (*e.g*., proton pump inhibitors, nonsteroidal anti-inflammatory medications, and antibiotics).^14,15^ Postrenal AKI from urinary obstruction is common in patients with genitourinary cancers and bulky intra-abdominal masses (*e.g*., ovarian cancer and lymphoma). Finally, some elevations in serum creatinine do not reflect true kidney injury or impairment; such cases of pseudo-AKI are attributed to off-target effects of chemotherapeutics on the tubular secretion of creatinine.

**Table 1:** AKI caused by antineoplastic therapies

Drug Class	Most Common Kidney Lesions	Biomarkers
Platinum-based agents	ATN, chronic interstitial fibrosis	Urine sediment, NGAL, KIM-1, TIMP-2, IGFBP7
Antimetabolites	Crystal-induced nephropathy, tubular atrophy interstitial fibrosis, TMA	Urine sediment examination (crystals), blood smear, haptoglobin, LDH, reticulocyte count, UPCR
Alkylating agents	ATN, hemorrhagic cystitis	Urine sediment, NGAL, KIM-1, TIMP-2, IGFBP7
BRAF inhibitors	ATN, AIN, pseudo-AKI (vemurafenib)	Urine sediment, cystatin C
Proteosome inhibitors	ATN, TMA, tumor lysis syndrome	NGAL, KIM-1, blood smear, haptoglobin, LDH, reticulocyte count, UPCR
Tyrosine kinase inhibitors	ATN, pseudo-AKI, TMA, FSGS	NGAL, KIM-1, cystatin C, UPCR
Antiangiogenesis (VEGF and VEGFR inhibitors)	TMA, podocytopathy	Blood smear, haptoglobin, LDH, reticulocyte count), UPCR
ICIs	AIN (most common)	Urine sediment examination, uRBP/Cr ratio, urine CXCL9, sIL-2R, CRP
EGFR inhibitors	Electrolyte disorders, glomerulopathy	Urine electrolytes, UPCR
mTOR inhibitors	ATN, FSGS	UPCR, NGAL, KIM-1
CDK4/6 inhibitors	Pseudo-AKI	Cystatin C
PARP inhibitors	Pseudo-AKI	Cystatin C

AIN, acute interstitial nephritis; ATN, acute tubular necrosis; BRAF, V-raf murine sarcoma viral oncogene homolog B; CDK4/6, cyclin-dependent kinase 4/6; Cr, creatinine; CRP, C reactive protein; CXCL9, C-X-C motif ligand 9; EGFR, EGF receptor; ICI, immune checkpoint inhibitor; mTOR, mammalian target of rapamycin; NGAL, neutrophil gelatinase-associated lipocalin; KIM-1, kidney injury molecule-1; IGFBP7, IGF-binding protein 7; LDH, lactate dehydrogenase; PARP, poly (ADP-ribose) polymerase inhibitor; sIL-2R, soluble IL-2 receptor; TIMP-2, tissue inhibitor metalloproteinase-2; TMA, thrombotic microangiopathy; UPCR, urine protein–creatinine ratio; uRBP, urine retinol-binding protein; VEGF, vascular endothelial growth factor; VEGFR, vascular endothelial growth factor receptor.

Establishing the underlying etiology of AKI in patients with cancer has important implications for both cancer and kidney care. For example, those with prerenal AKI may be treated with volume repletion, those with ATN with discontinuation of nephrotoxic medications, and those with AIN with withdrawal of culprit medications and use of immunosuppression. Accurate diagnosis is essential to facilitate prompt treatment and resolution of AKI and to prevent the risks of unnecessary cancer treatment interruptions and additional therapies.

## Traditional Blood and Urine Biomarkers of AKI

### Estimation of GFR

Accurate assessment of GFR is especially important in patients with cancer who may require dose adjustment of chemotherapies, determination of eligibility for clinical trials, and serial monitoring for nephrotoxicity. GFR is routinely estimated using serum creatinine, which is produced by the metabolism of creatinine phosphate in muscles. Owing to its simplicity of measurement and extensive validation, creatinine is the most widely used biomarker of kidney function and has become the gold standard for estimation of GFR using the 2021 race-free CKD Epidemiology Collaboration equation.^16^ However, patients with cancer commonly have reduced muscle mass (sarcopenia), which leads to overestimation of GFR when relying on creatinine-based equations.^17–19^

As such, there is growing utilization of cystatin C for GFR estimation, especially in patients with sarcopenia. Cystatin C is a small protease inhibitor that is produced at a constant rate by nucleated cells and is filtered, but neither secreted nor reabsorbed. It is significantly less dependent on muscle mass, although acute inflammation, large tumor cell burden, thyroid disease, and corticosteroid use can falsely increase cystatin C levels.^20^ Although widespread use of cystatin C has been hindered by prolonged turnaround time,^21^ use of GFR estimating equations that incorporate both cystatin C and creatinine is generally preferred in patients with cancer.^22^

Cystatin C may be particularly useful as an alternative to creatinine-based measurements when there is suspicion for pseudo-AKI or elevation in creatinine occurring due to the off-target effects of cancer therapies on organic cation transporters and multidrug and toxin extrusion transporters in the tubular creatinine secretion pathway.^23,24^ By inhibiting these transporters, targeted therapies, such as cyclin-dependent kinase 4/6 inhibitors, poly-ADP ribose polymerase inhibitors, tyrosine kinase inhibitors, and certain V-raf murine sarcoma viral oncogene homolog B inhibitors (vemurafenib), may lead to increases in creatinine in the absence of a true change in GFR.^25–30^ In this scenario, cystatin C could provide a more accurate estimation of GFR, although further studies are necessary to clarify non-GFR determinants of cystatin C in patients with cancer.

### Urinalysis and Urine Sediment Examination

Classic urine sediment findings that characterize different AKI etiologies are shown in Figure 1. A bland urine sediment with hyaline casts is consistent with prerenal azotemia. Presence of granular casts can help to diagnose ATN and determine the prognosis of AKI in patients with ATN.^31,32^ Although white blood cell casts are considered a specific finding in AIN, this test has poor sensitivity because <15% of patients with biopsy-proven AIN demonstrate this finding.^33^ In addition, urine eosinophils are no longer recommended for diagnosis of AIN because of poor accuracy.^34,35^ Presence of dysmorphic red blood cells and red blood cell casts can suggest GN, which can be a paraneoplastic phenomenon of solid organ cancers and a manifestation of monoclonal gammopathy of renal significance. Presence of crystals in the urine sediment can suggest crystalline nephropathy, as with high-dose methotrexate.

Taken together, although the urine sediment can be a valuable diagnostic test, the performance of the urine sediment examination is limited by interobserver variability,^36^ low sensitivity and specificity, and a poor negative likelihood ratio for the diagnosis of many etiologies of AKI.

### Urine Protein Quantification

Low-grade or tubular proteinuria commonly accompanies AKI; higher degrees of proteinuria accompany AKI when endothelial cells or podocytes are injured. The differential diagnosis for proteinuria is broad, including glomerular and tubular lesions, as well as overflow proteinuria, due to paraproteinemia. Albuminuria, which can be detected using dipstick tests and quantified using urine albumin–creatinine ratio on spot or 24-hour urine collection, can reflect glomerular and proximal tubular involvement. Proteinuria other than albuminuria can also be quantified using urine protein–creatinine ratio on spot or 24-hour urine collection, and monoclonal protein can be identified and quantified on urine protein electrophoresis and immunofixation.

The urine albumin–protein ratio (UAPR) may help determine the cause of AKI. Glomerular diseases (*e.g*., paraneoplastic minimal change or membranous) are associated with albumin-dominant proteinuria (UAPR >0.4). In tubular diseases, the injured proximal tubular cells fail to reabsorb low mol wt proteins; this can be quantified by an elevated urine protein–creatinine ratio with normal or only slightly elevated urine albumin–creatinine ratio. Of note, a low UAPR <0.4 is 88% sensitive and 99% specific for tubular disease.^37^

Finally, a large urine protein gap can be detected in patients with myeloma when extremely high levels of Bence Jones proteins are the cause of high-grade proteinuria with normal or only mildly increased urinary albumin and can be a diagnostic clue that a patient has myeloma kidney.^37,38^ Alternatively, an elevated urine protein gap could suggest the presence of lysozyme, which is an approximately 15 kDa protein produced by malignant mononuclear cells that are filtered in the glomerulus and reabsorbed by the proximal tubule, where it may cause tubulopathy and AKI.^39^ Although testing for urinary lysozyme levels is not universally available, urinary lysozyme may be detected on urine protein electrophoresis/immunofixation, and serum lysozyme is often above the upper limit of normal.

### Urine Electrolytes

The measurement of urine electrolytes has utility in patients with suspected tubular electrolyte wasting. Proximal tubulopathy may be caused by chemotherapies, including cisplatin, carboplatin, ifosfamide, and lenalidomide,^40^ and may also occur due to the direct effects of paraprotein disease on the proximal tubule, as in myeloma/light-chain disease and amyloidosis.^41^ Proximal tubulopathy manifests in Fanconi syndrome, characterized by hypokalemia, hypophosphatemia, hypouricemia, and acidemia due to urinary electrolyte losses, as well as aminoaciduria and proteinuria.

Isolated hypomagnesemia can be observed in patients receiving platinum-based chemotherapy and EGF receptor inhibitor use.^42,43^

## Imaging for Obstructive AKI and AIN in Patients with Cancer

Especially in genitourinary tract cancers and malignancies with bulky retroperitoneal disease with lymphadenopathy, ultrasound offers a noninvasive method to visualize the bladder and detect urinary outlet obstruction, as well as upper tract obstruction, by identifying hydroureter or hydronephrosis. While ultrasound can provide detailed images without exposing patients to ionizing radiation,^44^ cross-sectional imaging may also be necessary to evaluate the cause and location of urinary obstruction and to evaluate for infiltrative disease.

There are emerging data to suggest that cross-sectional imaging could assist in the diagnosis of AIN. A study of 34 patients with biopsy-proven ICI-AIN found that 15 patients (44%) had detectable imaging abnormalities on computed tomography (CT) scan or CT with positron emission tomography (PET) scan, reporting either increased total kidney volume or perinephric stranding.^45^ Other studies using PET/CT have shown that the mean standardized uptake value of radiotracer increases in tissues affected by immune-related adverse events, including the kidney^46–48^; importantly, this requires a baseline PET/CT for comparison, which is not always available. Furthermore, these studies are limited by small sample size and lack robust control populations of patients taking ICIs for comparison. Further studies will be required to standardize and validate the use of PET/CT to differentiate AIN and ATN in patients with cancer, especially given the inherent variability of PET/CT technical parameters (*e.g*., time to imaging after radiotracer injection, burden of metastatic disease uptaking radiotracer, radiotracer dose, and fasting state).^49^

Taken together, the high cost and lack of sensitivity and specificity of imaging to diagnose the cause of AKI in patients with cancer pose limitations to widespread use to differentiate AIN from ATN. Further research will be necessary to justify the routine use of imaging modalities as diagnostic tools for intrinsic AKI.

## Novel Blood and Urine Biomarkers

### Biomarkers of AIN

**Figure 2:**
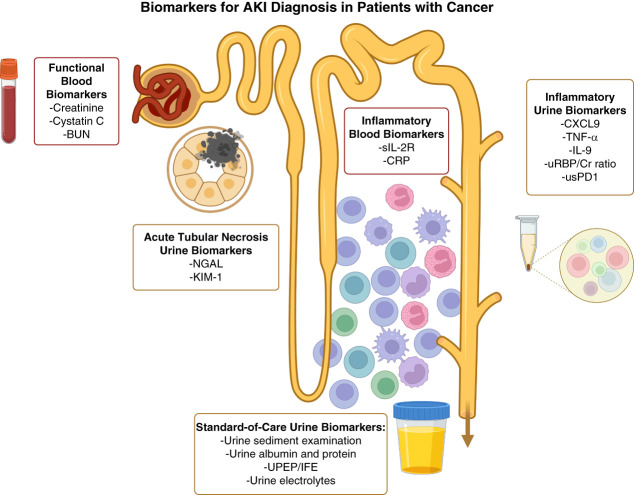
**Biomarkers for AKI diagnosis in patients with cancer.** Traditional biomarkers for AKI diagnosis in patients with cancer include functional blood biomarkers, such as creatinine, cystatin C, and BUN, as well as standard-of-care urine biomarkers, including urine albumin and protein, UPEP, and urine electrolytes. Novel biomarkers include urine biomarkers for ATN, inflammatory blood biomarkers, and inflammatory urine biomarkers, the most promising of which is CXCL9. Cr, creatinine; CRP, C-reactive protein; CXCL9, C-X-C motif ligand 9; IFE, immunofixation electrophoresis; KIM-1, kidney injury molecule-1; NGAL, neutrophil gelatinase-associated lipocalin; sIL-2R, soluble IL-2 receptor; TNF-*α*, TNF alpha; UPEP, urine protein electrophoresis; uRBP, urine retinol-binding protein; usPD1, urine soluble programmed death-1 protein. Created in BioRender. Mistry *et al.*^8^ BioRender.com/m94y654.

Despite currently clinically available tests, there are still notable challenges in determining the cause of AKI, which highlights the need for novel biomarkers (Figure 2). Numerous recent studies have focused on identifying biomarkers of ICI-AIN because the current clinically available noninvasive tests (*e.g*., urinalysis, sediment, and kidney ultrasound) have poor accuracy in distinguishing ICI-AIN from other causes of AKI in patients on ICIs.^50–53^ Patients with ICI-AIN require temporary or permanent cessation of therapy and are treated with corticosteroids; thus, inaccurate assumption of ICI-AIN diagnosis can lead to withdrawal of lifesaving cancer therapy and use of toxic immunosuppressive medications, whereas missed AIN diagnosis could lead to permanent kidney damage.

The most promising urine biomarker is C-X-C motif ligand 9 (CXCL9), a chemokine that is induced by IFN-*γ*, which has been identified to be upregulated in AIN by several independent research groups. Our group identified urine CXCL9 as the key diagnostic biomarker for AIN through urine proteomics showing that CXCL9 levels were higher in urine and kidney tissue of patients with AIN compared with controls.^54^ The area under the curve (AUC) of CXCL9 for AIN diagnosis was 0.94 in external validation cohorts, and CXCL9 improved the AUC for AIN diagnosis over a model of currently available clinical tests for AIN, as well as over clinicians' prebiopsy suspicion for AIN.

There is a significant body of evidence supporting the biological importance of IFN-*γ* and associated chemokines in the pathogenesis of immune-related adverse events in multiple organs. One study showed that among patients with cancer on ICIs, those whose T cells proliferate and produce CXCL9 within 1–2 weeks of initiating therapy later experience immune-related adverse events, including ICI-AIN.^55^ Moreover, gene expression profiling of tissue from patients with ICI-AIN reveal CXCL9 and other IFN-*γ* downstream chemokines as some of the top differentially expressed genes.^56^ A customized urinary 12-chemokine panel based on these differentially expressed genes showed close correlation with tissue immune signatures, suggesting the potential for this panel to be a novel biomarker for differentiating ICI-AIN from other causes of AKI (AUC >0.75).^56^

Two urine biomarkers that have been associated with biopsy-proven AIN in the noncancer setting include TNF-*α* and IL-9, which are also markers of steroid responsiveness (patients with higher levels of urine IL-9 tended to have a more favorable response to steroid therapy).^57,58^ In the cancer setting, the promise of urinary TNF-*α* as a biomarker of ICI-AIN was demonstrated in a study of 24 patients on ICI therapy, 14 of whom had biopsy-proven ICI-AIN or clinically adjudicated ICI-AIN.^59^ Urinary TNF-*α* was increased in patients with ICI-AIN, and urinary levels positively correlated with tissue TNF-*α* for those patients whose kidney tissue was available, suggesting that urinary TNF-*α* originates from the kidneys. Furthermore, the addition of TNF-*α* and IL-9 to a model including serum creatinine, BUN–creatinine ratio, dipstick specific gravity, and dipstick protein improved the AUC for diagnosis of AIN over clinicians' prebiopsy suspicion for AIN.^50^

Another study showed patients with biopsy-proven or clinically adjudicated ICI-AIN exhibited higher blood levels of soluble IL-2 receptor (sIL-2R), as compared with ICI-treated controls without AKI and patients with hemodynamic AKI not taking ICIs.^60^ sIL-2R is a marker of T-cell dysregulation and may rise in the setting of inflammatory processes, including ICI-AIN, sepsis, and extrarenal immune-related adverse events. A sIL-2R cutoff of 1.75-fold upper limit of normal showed optimal specificity (100%) and sensitivity (81%) and an AUC >0.96 when using either a comparator group without sepsis or other immune-related adverse events as a control.

Another recent study identified urine soluble programmed death-1 protein as a biomarker of ICI-AIN, noting that the ratio of urine soluble programmed death-1 protein levels in ICI-AIN to other causes of AIN was 2.7 (AUC, 0.83) for diagnosis of AIN versus ATN.^61^

Roles for urine retinol-binding protein–creatinine ratio and urinary monocyte chemoattractant protein 1 as biomarkers of proximal tubular injury have been established in the noncancer setting.^62–64^ A retrospective study in 50 patients with cancer taking ICIs revealed that urine retinol-binding protein/Cr ratio and serum C-reactive protein were higher in the ICI-AKI group compared with the non–ICI-AKI group.^65^ There are some recent data to suggest that among 39 patients with cancer treated with ICIs and/or platinum derivatives, urinary monocyte chemoattractant protein 1 is elevated in those with ICI-AIN as opposed to those with ATN and correlates with serum C-reactive protein.^66^

In addition to urinary biomarkers, histopathological markers, such as programmed death-ligand 1 on renal tubular epithelial cells and programmed cell death protein 1 on interstitial cells, correlate with ICI-AIN as opposed to other causes of AKI.^67,68^ In addition, there is a growing body of evidence that implicates specific T-cell populations in the pathogenesis of ICI-AIN with studies to date using multiplexed expression analysis techniques, such as imaging mass cytometry.^59^ Further comprehensive studies using single-cell RNA sequencing and spatial transcriptomics technologies will be required to fully elucidate the cellular drivers of ICI-AIN and to nominate candidate biomarkers for further development.

### Biomarkers of ATN

Numerous biomarkers of ATN have been identified, although they have yet to be deployed widely in the clinical setting for diagnosis of cancer-related AKI. Neutrophil gelatinase-associated lipocalin (NGAL) is a protein in neutrophil granules that is upregulated ten-fold within the first few hours of ischemic kidney injury in mouse models^69^ and can be detected in the kidney and urine within 3 hours of cisplatin administration.^70^ NGAL was recently cleared by the US Food and Drug Administration for AKI risk assessment in young patients aged 3 months to 21 years, and as such, we anticipate increasing utilization of this test to differentiate ATN from hemodynamic AKI.

Kidney injury molecule-1 (KIM-1) is a biomarker of ischemic and toxic kidney injury.^71^ The ectodomain of KIM-1 is shed from cells into urine in rodents and humans after proximal tubular injury.^72–74^ A prospective study of pediatric patients receiving cisplatin found higher urine NGAL and KIM-1 concentrations in patients with AKI as compared with those without AKI.^75^

There is growing interest in early detection of AKI before serum creatinine rises, which may enable timely and effective intervention in the face of impending injury. Markers of cell cycle arrest, such as tissue inhibitor metalloproteinase-2 and IGF-binding protein 7, have been studied in the noncancer population as indicators of subtle changes in kidney function that predict the onset of AKI (AUC, 0.94 for a combination of tissue inhibitor metalloproteinase-2 and IGF-binding protein 7).^76^ Further studies are required to validate their predictive ability in patients with cancer.

## Conclusions and Future Directions

There remains a significant unmet need for improved diagnostics in AKI for patients with cancer, both to facilitate early diagnosis and intervention to prevent cancer treatment interruptions, as well as to enable risk stratification to guide prognostication and management. The heterogeneity of anticancer drugs and their nephrotoxic mechanisms will require an array of biomarkers that not only exhibit sensitivity and specificity in detecting AKI across diverse patient populations, but also reflect the underlying pathophysiology of AKI and provide insights into the cellular and molecular mechanisms of kidney injury and repair. In addition to the intrinsic challenges of biomarker discovery and validation, there are numerous technical, regulatory, and systemic hurdles that pose barriers to the translation of novel biomarkers into widely used diagnostic tests. Addressing these challenges will require interdisciplinary collaboration, innovative approaches, and sustained investment and interest in biomarker research and development.

## Supplementary Material
