# Supplementary material for: Novel Biomarkers and Imaging Tests for Acute Kidney Injury Diagnosis in Patients with Cancer
Source: Kidney360. 2024 Nov 21;6(1):167–74. doi: 10.34067/KID.0000000660 (PMC11793192; doi:10.34067/KID.0000000660)
Supplement: Supplementary file 1 [file kidney360-6-167-s001.pdf]

ASN American Society of Nephrology
JASN Journal of the American Society of Nephrology
KIDNEY360 Accessing Our World From Every Angle
CJASN Clinical Journal of the American Society of Nephrology

ASN Journal Disclosure Form

As per ASN journal policy, I have disclosed any financial relationships or commitments I have held in the past 36 months as included below. I have listed my Current Employer below to indicate there is a relationship requiring disclosure. If no relationship exists, my Current Employer is not listed.

S. Mejia reports the following:
Employer: Massachusetts General Brigham

I understand that the information above will be published within the journal article, if accepted, and that failure to comply and/or to accurately and completely report the potential financial conflicts of interest could lead to the following: 1) Prior to publication, article rejection, or 2) Post-publication, sanctions ranging from, but not limited to, issuing a correction, reporting the inaccurate information to the authors' institution, banning authors from submitting work to ASN journals for varying lengths of time, and/or retraction of the published work.

Name: Sherley M Mejia
Manuscript ID: K360-2024-000583R2
Manuscript Title: Novel biomarkers and imaging tests for AKI diagnosis in patients with cancer
Date of Completion: November 15, 2024
Disclosure Updated Date: November 15, 2024

ASN Journal Disclosure Form

As per ASN journal policy, I have disclosed any financial relationships or commitments I have held in the past 36 months as included below. I have listed my Current Employer below to indicate there is a relationship requiring disclosure. If no relationship exists, my Current Employer is not listed.

K. Mistry reports the following:
Employer: Brigham and Women's Hospital; Beth Israel Deaconess Medical Center; Ownership Interest: Vertex Pharmaceuticals; CRISPR therapeutics; and Other Interests or Relationships: American Society of Nephrology Continuous Professional Development Committee - Fellow Member; American Kidney Fund - Recipient of Clinical Scientist in Nephrology Award.

I understand that the information above will be published within the journal article, if accepted, and that failure to comply and/or to accurately and completely report the potential financial conflicts of interest could lead to the following: 1) Prior to publication, article rejection, or 2) Post-publication, sanctions ranging from, but not limited to, issuing a correction, reporting the inaccurate information to the authors' institution, banning authors from submitting work to ASN journals for varying lengths of time, and/or retraction of the published work.

Name: Kavita Mistry
Manuscript ID: K360-2024-000583R1
Manuscript Title: Novel biomarkers and imaging tests for AKI diagnosis in patients with cancer
Date of Completion: October 22, 2024
Disclosure Updated Date: October 22, 2024

ASN American Society of Nephrology
JASN Journal of the American Society of Nephrology
KIDNEY360 Accessing Our World From Every Angle
CJASN Clinical Journal of the American Society of Nephrology

ASN Journal Disclosure Form

As per ASN journal policy, I have disclosed any financial relationships or commitments I have held in the past 36 months as included below. I have listed my Current Employer below to indicate there is a relationship requiring disclosure. If no relationship exists, my Current Employer is not listed.

D. Moledina reports the following:
Employer: Yale University School of Medicine; Consultancy: BioHaven, Inc.; Ownership Interest: Predict AIN, LLC; Research Funding: NIDDK; Honoraria: Healthcentral; British medical journal; Patents or Royalties: DGM is a coinventor of the pending patent application "Methods and Systems for Diagnosis of Acute Interstitial Nephritis"; and Advisory or Leadership Role: ASN journals, editorial board member.

I understand that the information above will be published within the journal article, if accepted, and that failure to comply and/or to accurately and completely report the potential financial conflicts of interest could lead to the following: 1) Prior to publication, article rejection, or 2) Post-publication, sanctions ranging from, but not limited to, issuing a correction, reporting the inaccurate information to the authors' institution, banning authors from submitting work to ASN journals for varying lengths of time, and/or retraction of the published work.

Name: Dennis G. Moledina
Manuscript ID: K360-2024-000583R1
Manuscript Title: Novel biomarkers and imaging tests for AKI diagnosis in patients with cancer
Date of Completion: October 14, 2024
Disclosure Updated Date: October 14, 2024

ASN American Society of Nephrology
JASN Journal of the American Society of Nephrology
KIDNEY360 Accessing Our World From Every Angle
CJASN Clinical Journal of the American Society of Nephrology

ASN Journal Disclosure Form

As per ASN journal policy, I have disclosed any financial relationships or commitments I have held in the past 36 months as included below. I have listed my Current Employer below to indicate there is a relationship requiring disclosure. If no relationship exists, my Current Employer is not listed.

D. Moreno has nothing to disclose.

I understand that the information above will be published within the journal article, if accepted, and that failure to comply and/or to accurately and completely report the potential financial conflicts of interest could lead to the following: 1) Prior to publication, article rejection, or 2) Post-publication, sanctions ranging from, but not limited to, issuing a correction, reporting the inaccurate information to the authors' institution, banning authors from submitting work to ASN journals for varying lengths of time, and/or retraction of the published work.

Name: Daiana R. Moreno
Manuscript ID: K360-2024-000583R2
Manuscript Title: Novel biomarkers and imaging tests for AKI diagnosis in patients with cancer
Date of Completion: November 15, 2024
Disclosure Updated Date: November 15, 2024

ASN
American Society of Nephrology
JASN
Journal of the American Society of Nephrology
KIDNEY360
Accessing Our World From Every Angle
CJASN
Clinical Journal of the American Society of Nephrology

ASN Journal Disclosure Form

As per ASN journal policy, I have disclosed any financial relationships or commitments I have held in the past 36 months as included below. I have listed my Current Employer below to indicate there is a relationship requiring disclosure. If no relationship exists, my Current Employer is not listed.

S. Sadarangani has nothing to disclose.

I understand that the information above will be published within the journal article, if accepted, and that failure to comply and/or to accurately and completely report the potential financial conflicts of interest could lead to the following: 1) Prior to publication, article rejection, or 2) Post-publication, sanctions ranging from, but not limited to, issuing a correction, reporting the inaccurate information to the authors' institution, banning authors from submitting work to ASN journals for varying lengths of time, and/or retraction of the published work.

Name: Sagar S. Sadarangani
Manuscript ID: K360-2024-000583R1
Manuscript Title: Novel biomarkers and imaging tests for AKI diagnosis in patients with cancer
Date of Completion: October 16, 2024
Disclosure Updated Date: October 16, 2024

ASN
American Society of Nephrology
JASN
Journal of the American Society of Nephrology
KIDNEY360
Accessing Our World From Every Angle
CJASN
Clinical Journal of the American Society of Nephrology

ASN Journal Disclosure Form

As per ASN journal policy, I have disclosed any financial relationships or commitments I have held in the past 36 months as included below. I have listed my Current Employer below to indicate there is a relationship requiring disclosure. If no relationship exists, my Current Employer is not listed.

M. Sise reports the following:
Employer: Massachusetts General Hospital; Consultancy: Vera, Travere, Calliditas, Mallinckrodt, Novartis, Otsuka, Alpine Immune sciences, RelayTx (self). Emed, X-Biotix (Spouse); Ownership Interest: X-Biotix (spouse); Research Funding: Angion, Otsuka, Gilead, Cabaletta, Novartis, Roche/Genetech, Merck; Patents or Royalties: US 11,441,196 B2 : Roby Bhattacharyya; RIBOSOMAL RIBONUCLEIC ACID HYBRIDIZATION FOR ORGANISM IDENTIFICATION; and Advisory or Leadership Role: X-Biotix (spouse).

I understand that the information above will be published within the journal article, if accepted, and that failure to comply and/or to accurately and completely report the potential financial conflicts of interest could lead to the following: 1) Prior to publication, article rejection, or 2) Post-publication, sanctions ranging from, but not limited to, issuing a correction, reporting the inaccurate information to the authors' institution, banning authors from submitting work to ASN journals for varying lengths of time, and/or retraction of the published work.

Name: Meghan E. Sise
Manuscript ID: K360-2024-000583R2
Manuscript Title: Novel biomarkers and imaging tests for AKI diagnosis in patients with cancer
Date of Completion: November 15, 2024
Disclosure Updated Date: November 15, 2024
